# Challenges in Human Skin Microbial Profiling for Forensic Science: A Review

**DOI:** 10.3390/genes11091015

**Published:** 2020-08-28

**Authors:** Ana Neckovic, Roland A. H. van Oorschot, Bianca Szkuta, Annalisa Durdle

**Affiliations:** 1 School of Life and Environmental Sciences, Deakin University, Geelong 3220, Australia; b.szkuta@deakin.edu.au (B.S.); a.durdle@deakin.edu.au (A.D.); 2 Office of the Chief Forensic Scientist, Victoria Police Forensic Services Centre, Macleod 3085, Australia; roland.vanoorschot@police.vic.gov.au; 3 School of Molecular Sciences, La Trobe University, Bundoora 3086, Australia

**Keywords:** human microbiome, microbial profiling, forensic science, microbial transfer, forensic limitations

## Abstract

The human microbiome is comprised of the microbes that live on and within an individual, as well as immediately surrounding them. Microbial profiling may have forensic utility in the identification or association of individuals with criminal activities, using microbial signatures derived from a personal microbiome. This review highlights some important aspects of recent studies, many of which have revealed issues involving the effect of contamination of microbial samples from both technical and environmental sources and their impacts on microbiome research and the potential forensic applications of microbial profiling. It is imperative that these challenges be discussed and evaluated within a forensic context to better understand the future directions and potential applications of microbial profiling for human identification. It is necessary that the limitations identified be resolved prior to the adoption of microbial profiling, or, at a minimum, acknowledged by those applying this new approach.

## 1. Introduction

The microbiome is often defined as the total genetic content of a microbial community, but the term may also be used to refer to the physical microbial community within a given environment [1]. The human microbiome, more specifically, refers to the microbial communities that are human-associated, living both on and within individuals and immediately surrounding them [2]. Research has revealed that human microbiomes are highly individualised [3,4]; human gut microbial compositions, for example, may reveal microbiota that are intrinsically linked to factors such as an individual’s diet [5], lifestyle [6] and biogeography [7]. Profiling of human microbiomes has increased over recent years, particularly within the health sciences, due to a combination of the decreasing costs associated with sequencing, and the accessibility of bioinformatics protocol knowledge and software [8]. Additionally, research interest surrounding the role and significance of microbiomes in human health and disease has increased dramatically within the last decade [9]; however, the field today remains largely composed of descriptive, rather than functional, analyses of microbiomes [10].

Microbial profiling is an emerging area of interest within forensic science, now included in the expanded definition for ‘microbial forensics’ [11], which is largely tasked with researching the potential to identify individuals, or to associate them to items and/or environments through the analysis of distinguishing microbial features (i.e., bacteria, fungi or viruses) within their microbiomes. Recent studies have revealed that individuals demonstrate highly personalised microbial signatures within their microbiomes [12,13,14,15,16]; these microbial signatures are thought to have potential forensic utility, to identify or associate an individual with a criminal activity. Indeed, nuclear DNA (nDNA) profiling currently serves this forensic purpose and is considered the gold standard within the industry for human identification [17]; however, microbial profiling may complement existing techniques in instances when nDNA retrieved from a crime scene or evidentiary item is of low quantity or poor quality.

Human microbiomes have been sampled and characterised from multiple body sites including the skin [13], gut [18,19], oral [20] and upper respiratory tracts [21], pubic regions [12,22], hair [22,23,24], and even ocular surfaces [25]. While it is understood that forensic microbiome samples of interest may involve other biological sample matrices, including body fluids (i.e., saliva, vaginal secretions and blood [26,27]), this review predominantly discusses research advances concerned with human skin microbiomes and their potential forensic application. Human skin microbiomes have an interesting potential to be utilised as trace evidence due to factors involving shedding propensities (e.g., skin squames [28]), body site-driven differences in microbial community compositions [29], and the sheer abundance of microbial cells by comparison to human somatic cells, particularly bacterial cells [30]. These skin-associated bacteria are deposited within, and can colonise on built environments [31,32,33,34,35,36,37], which suggests that human microbiomes may be more readily retrieved from crime scenes compared to nDNA, providing possible alternate investigative leads.

Within the body of research investigating the potential forensic utility of skin microbiomes as trace evidence, a significant proportion of studies involve the analysis of bacterial communities through targeted sequencing of the 16S ribosomal RNA (rRNA) region [12,15,16,34,38,39,40,41,42,43,44,45,46,47]. This region is conserved within bacteria, but harbours enough interspecific variation to allow different bacterial species to be discerned through sequencing [48]. Other regions of interest within human microbiomes, or mycobiomes (i.e., fungal communities), include the 18S rRNA, and the Internal Transcribed Spacer (ITS) region [49]. It is important to note that human skin microbiomes have been assessed for forensic utility through shotgun metagenomic sequencing; this technique has greater discriminatory power in comparison to microbial community profiling, and this provides a positive, substantiated direction forward for microbial forensics [13,14,50]. This review, however, predominantly discusses the forensic applications of human microbial profiling via 16S rRNA bacterial profiling. It should be noted that the review was not conducted as a systematic review; studies within this limited body of knowledge concerning microbial profiling of human microbiomes, involving this methodology of analysis for forensic purposes, were included and discussed. Although the review intends to capture the bulk of papers across various fields, it is not exhaustive of all related publications. Some commentary on microbial profiling for forensic purposes is also provided more generally, irrelevant of the methodology of analysis. The purpose of this review is to highlight and discuss potential limitations associated with the forensic application of human microbiome analyses, and in doing so, provide further direction for this emerging area of interest whereby these limitations are acknowledged. The authors intend for this review to act as a vehicle to facilitate discussion among the forensic community, in order to flag issues that may occur when implementing such an approach for forensic human identification/association. The scope of this review will include an overview of how microbial profiling is performed, the factors to be considered for the forensic utility of microbiomes including sample collection and sources of contamination, and other considerations, which may be unique challenges to microbial forensics. A summary of the potential advantages and the associated limitations/challenges to be addressed for microbial profiling for forensic purposes has also been included for further consideration (Table 1). It is hoped that through the identification and discussion of these limitations, future research claims pertaining to forensic applications of microbial profiling will exercise restraint in overstating its potential forensic value.

## 2. Microbial Profiling Overview

Microbial communities, whether sampled from human or environmental sources, are often retrieved through swabbing, commonly using cotton swabs [15,35,51,52,53]. Following this, the DNA of both non-specific targets and the microbial communities is extracted using either standard or optimised DNA extraction protocols, through the use of commercially available DNA isolation kits. The samples then undergo amplicon library preparation, involving ligation of primers and PCR amplification, aimed at targeting specific genetic markers within microbial communities, such as the 16S rRNA for bacterial communities [10]. Following the addition of sample indices (barcodes) and amplification, amplicon libraries are multiplexed (pooled) and the region(s) of interest are sequenced, typically on Illumina platforms [54]. Sequenced reads must then be assigned to each individual sample (demultiplexed) before further computational analyses are carried out. Several bioinformatics pipelines exist for microbiome data processing, refining raw sequences into informative visualisations; two of the most commonly used are Quantitative Insights Into Microbial Ecology (QIIME, or more recently, QIIME2 [55]) and Mothur [56]. Microbial sequences may first be quality filtered using Deblur or the Divisive Amplicon Denoising Algorithm (DADA2) [57] to infer amplicon sequence variants (ASVs). Taxonomic analyses may then be achieved by mapping these variants using a Naïve Bayes classifier, against commonly used reference databases including Greengenes [58] and SILVA [59]. Typically, analyses involving the microbiomes of individuals will include assessments on α (within) and β (between) diversity analyses, using a variety of distance metrics including Unweighted Unifrac [60]. Metrics such as Unweighted Unifrac focus on the presence/absence of distinguishing features (e.g., ASVs) within samples and are useful for distinguishing microbial communities between samples using numeric distances. This information may be visually interrogated through Principal Coordinates Analysis (PCoA) [61], whereby samples, and their distances relative to each other, are arranged within a 2D or 3D space; samples that appear to cluster are considered to be more similar in their microbial compositions than samples that are further apart. Many of the publications that have highlighted the potential forensic utility of human microbiomes via a 16S rRNA approach have utilised these types of analyses to associate individuals with the microbiomes of items they have touched [15,16], people they cohabitate with [12,62], or places they live/work in [33,42,52].

## 3. Forensic Microbiome Analyses: Elements to Be Considered

### 3.1. Microbiome Transfer and Persistence

Human DNA transfer, persistence, prevalence and recovery (DNA-TPPR) are active areas of research within forensic biology concerned with providing empirical data to inform probabilities used to calculate the likelihood of the DNA evidence given alternate propositions surrounding the activities of interest [63,64]. The need for human DNA transfer research has been driven by both an increase in the sensitivity of human DNA detection and profiling techniques, and challenges arising during criminal trial proceedings. These challenges typically question the mechanisms by which a suspect’s DNA may be deposited on an evidentiary item or at a crime scene, often suggesting that an indirect transfer event may have occurred in lieu of direct contact [64,65]. Given that the human microbiome may potentially be utilised to associate or identify individuals for forensic investigation, the need for TPPR research is also required in relation to microbial DNA associated with human microbiomes. However, further complications with such research lie in the complexity of what constitutes a microbiome, as microbiomes involve ecological interactions [66] that demonstrate temporal shifts [67]. Human microbiomes may be transferred between cohabitating couples, family members and their pets [62,68], students who share dormitory rooms [34] and via direct and indirect mechanisms between non-cohabitating individuals’ hands [69]. Through the shedding of one’s skin-associated microbiota, which is collectively referred to as the ‘microbial cloud’ of an individual, human microbiomes may also be indirectly deposited into built environments [2,31]. While studies have assessed the direct transfer of human microbiomes, linking individuals to personal effects [16,70], office equipment [15], fabrics [44], and shared spaces/surfaces within homes (e.g., kitchen counters) [32], the persistence of a transferred microbiome over time is yet to be comprehensively understood. Some indicators within these previous studies are also somewhat contradictory, suggesting that microbial signatures may either persist on items within office or home environments over a short period of time [15,16], or, decay rapidly from the surfaces once the environment is no longer occupied [32]. This inconsistency may be due to how different surface types affect the persistence of microbiomes, including surfaces commonly encountered in a crime scene. However, there is limited information surrounding the persistence of microbiomes on different surface types, including the impact of additional factors such as pH, light, humidity/temperature and nutrient availability. Studying such factors, including microbial shifts between the time of deposition and collection of human-associated microbiota, may provide insight into the capacity for which human microbiomes may or may not be used to identify/associate an individual. This is particularly important, given that an individual’s reference microbiome may be collected at a much later stage in an investigation.

Studying the persistence of human microbiomes may be convoluted further by naturally existing temporal shifts in community compositions [67]; what may be a valid conclusion drawn from analysing the persistence of transferred microbiomes between one pair of individuals, for example, may not be valid for a different pair of individuals. This is due to human-associated microbiota existing on, within and around the body in a complex state of cooperation/competition with each other and their environments. Additionally, these microbial interactions are linked to many intrinsic and extrinsic factors, including nutrient availability [71], season [72], oral antibiotic use [73] and host diet [5], to name a few. When studied, microbiomes should be considered as ecosystems that require careful consideration from researchers regarding experimental design to limit confounding variables and enable the analysis of nuanced differences or associations within microbial community structures, or changes over time. Studies may indicate the persistence or absence of a particular microbe within the human microbiome and associate such presence/absence with a particular factor, such as lifestyle or dietary intake, indicated by detailed participant surveys, but this association does not correlate to proof [74]. It may be argued then that microbial profiling of individuals for forensic purposes may only indicate the potential association of specific microbiota to an individual’s microbiome but may not necessarily provide proof of the innate membership of that microbe to an individual’s microbiome. If the intended purpose of microbial identification would be to associate an individual with a criminal activity and/or inform investigations, this should raise concern.

Furthermore, such factors may also complicate assessments on whether, or how, microbial contamination within a forensic setting has occurred. While human nDNA databases may be used to indicate if staff contamination has occurred within a forensic setting (i.e., at a crime scene or within a forensic laboratory), the development of a staff microbiome database, and by extension a criminal microbiome database, may not have the same value, given that an individual’s microbiome has the potential to change in minor or significant ways over time, and the stability of human microbiomes is also considered to be an individual trait [75]. Though microbiomes of shared built environments have been studied, including office spaces [52] dormitories [76] and homes [32], no studies have addressed the microbial similarities of human microbiomes between staff members working in the same building versus a random population of individuals. These individuals sharing such spaces may have microbiomes that become homogenised or partially converged over an extended period of time, as has been demonstrated in the shared number of taxa observed in the microbiomes of cohabitating couples [77], families and pets [68]. These challenges may therefore limit the forensic utility of microbial profiling given the enormity of the task in developing, and maintaining, an accurate microbiome database.

### 3.2. Sample Collection and Storage

While microbiomes may be present and sampled from a variety of biological sample matrices such as body fluids [39], skin [78] and hair [23], microbiota may also be retrieved from surfaces onto which those biological samples are deposited, including personal effects [16]. This alludes to the potential for human skin-associated bacterial communities to be transferred during a routine item examination, despite the use of personal protective equipment (PPE) and/or adherence to standard operating procedures (SOPs) suitable for human DNA recovery. Indeed, recent preliminary research by the authors examining the microbiota on various surfaces within an examination room and on PPE during a mock evidence examination (unpublished data) has suggested the potential for evidentiary item microbiomes to be transferred to a forensic examiner or laboratory surfaces, or for them to be altered through the addition of the microbiome of the examiner or laboratory during a routine item examination. This demonstrates that if microbial profiling of human skin-associated bacteria were to be considered for forensic use, forensic scientists would also need to consider the risk of microbial contamination events within forensic settings (i.e., crime scene to laboratory) and use this information to design new, or optimise current, protocols for contamination minimisation to ensure that they are suitable for microbial profiling.

If microbial profiling were to be adopted as a forensic technique, forensic evidence collected from a crime scene that has been transported to a laboratory will need to be triaged appropriately prior to its examination to stabilise the microbial communities on or within the evidentiary items. Currently, there exist storage protocols to allow for sufficient recovery of good quality human nDNA from evidentiary items, such as the sealing of evidentiary items within the appropriate type of evidence bag or container (e.g., brown paper or plastic bags, nylon bags, tubes, envelopes) [79,80,81] and the storage of sealed evidentiary items within a fridge/freezer or cool, dry environments until required for analysis [82]. Additionally, since DNA evidence can be retrieved from crime scenes using cotton swabs [83], the drying of the swabs and/or aeration of swab containers is also critical to minimise the microbial degradation of nDNA during storage [80,84,85]. However, what may be appropriate protocol for the preservation of forensic biological evidence for human DNA typing may be inappropriate for the preservation and/or stabilisation of microbial communities. For example, the storage of evidentiary items within cool, dry environments may provide optimal conditions for specific microbes to proliferate while others become non-viable, or, exposure to low temperatures may induce changes in microbial growth and structure, as has been demonstrated for the skin-associated bacteria *Staphylococci* [86]. Additionally, drying of the cotton swabs or aeration of swab containers may allow external microbes to be introduced to the swab surface, while the evidentiary packaging itself may be the source of further microbial contamination. Standard protocols such as these may induce microbial community shifts over time, hence there would need to be information regarding the impacts of these various protocols made available through extensive research, and appropriate actions taken to adequately address any negative impacts. However, since an ‘expected’ microbial community composition cannot be established, in much the same way that ‘ground truth’ is rarely available in a criminal investigation, any microbial analyses following the collection and examination of an evidentiary item would need to be evaluated with extreme caution.

In addition to the optimisation of sample collection and storage protocols, it would also be necessary to monitor laboratory environments for background microbial DNA, as is currently conducted within molecular biology laboratories and ultra-clean laboratories utilised for ancient-DNA sampling [87] and low copy number DNA analysis. Since microbial profiling often returns non-negative results for control samples (e.g., negative extraction controls) [88,89,90], it may be difficult to discern if an environment utilised for evidence examination is indeed fit for purpose (i.e., void of background microbiomes) or, if the observed microbial contamination may originate solely from technical, rather than environmental sources. Given that such controls yield non-negative results, if microbial profiling were to be used in this form as a means of identifying or associating individuals for forensic purposes, it would be fair to assume that demonstrating the reliability or validity of such a technique to a trier of fact would be challenging due to the inherent presence of microbial contamination.

### 3.3. DNA Extraction and Sources of Contamination

A crucial component of microbial profiling is the extraction of the microbial DNA, to be utilised for the sequencing and interrogation of a microbial community of interest. However, there is an awareness of the impacts of DNA extraction on microbial profiling [51,91,92], particularly concerning the reproducibility of a sample profile when one type of kit and protocol is used [93], and the varying proportions of extracted microbial DNA from known compositions of mock microbial communities, when different extraction kits are used [91]. While extraction kits are benchmarked using positive controls containing mock microbial communities to determine extraction efficiencies, it cannot be guaranteed that those same kits released for commercial use would extract the correct proportions of an unknown microbial community [94]. It could be argued then, that the extraction of microbial DNA from an evidentiary sample may not accurately represent the microbial community of interest, the proportions of which may be intended to be used to determine how closely a sample resembles the microbiome of an individual, and therefore, the association of that individual with a criminal activity.

Furthermore, scientific reproducibility is an ongoing issue concerning microbiome research [95], whereby it is often challenging to reproduce microbial communities from samples of the same source using the same extraction methodology, or to reproduce the same result from one sample using different or updated extraction methodologies. This should raise doubt—or at least, concerns—surrounding the applicability of microbial profiling of individuals for forensic utility, given that an obtained result could be entirely dependent on the application of a particular extraction method, technician, laboratory environment or time from when a sample had been obtained. It should also be noted that extraction kits contain a background or reagent microbiome [90]; termed the ‘kitome’, this microbiome, derived solely from extraction kits, has the capacity to swamp genuine microbial signals within a sample, if that sample contains a low microbial load [88]. Low-biomass samples contain microbial DNA quantities similar to those of negative extraction (or blank) samples; therefore, any microbial DNA present within the sample can easily be outcompeted by external/contaminating microbial DNA [88]. Of most concern should be that kitomes can often contain the same microbes of interest within a target specimen. For example, *Cutibacterium acnes* (formely *Propionibacterium acnes* [96]) is both a genuine inhabitant of human skin microbiomes, and an extraction kit contaminant [97]. This particular bacterium, as well as *Corynebacterium*, has been previously detected within the negative extraction controls of microbiome studies [90], but are now also the dominant genera of a recently developed skin microbiome target panel, namely the ‘hidSkinPlex’ [50], which has been suggested for use in forensic human identification. Additionally, kitomes observed within negative extraction controls are often inconsistent between kits of the same type [98]. Even during the processing stages of negative extraction controls, within what may be considered a sterile environment, exogenous bacteria can be introduced to a sample simply through exposure to a laboratory atmosphere/environment, technician and/or equipment [94]. As a result, it may be difficult to determine whether a negative extraction control has been contaminated from microbes derived from the extraction kit or from an external source. Ultimately, it would be challenging for forensic scientists to filter genuine microbial signals compared to potential contaminants, as well as to identify and mitigate the source of such contaminants, which weakens the proposal for the forensic utility of microbial profiling.

### 3.4. Sequencing and Analysis

Further microbial contamination may occur during the sequencing process, even when negative extraction controls are employed and sequenced to potentially identify background contamination originating from extraction kits. Index-hopping can occur when non-ligated adapters for one sample bind to the free DNA of another sample on the same sequencing run; this can be especially problematic for low biomass samples, of which there may be a lack, or low DNA template [94]. Index-hopping is reported to occur in 1–10% of obtained sequencing data, but is dependent on the type of Illumina sequencer used [99]. Ultimately, this may result in the incorrect assignment of sequencing data from one sample to another, which in turn could create a microbial overlap between samples. This may be problematic, given a negative extraction control may reflect the same microbial profile as a sample of interest due to index-hopping effects [94]. Index-hopping can be mitigated using unique dual-indexing combinations [100], or, samples could be processed separately via multiple sequencing runs. However, samples separated by multiple sequencing runs may be susceptible to batch effects [101], which arise due to cross-contamination of microbial DNA between tubes/wells within a ‘batch’ of samples [88], inducing microbial overlaps. However, this increase in microbial community similarity between samples would be more problematic for forensic samples of different evidentiary origins (i.e., samples from different individuals, items or locations processed within the same run). While it is recommended that samples of different types be randomised across sequencing runs within microbiome studies [88] to reduce conflation of batch effects with true biological signals (e.g., time of sampling), forensic samples would ideally be processed separately. For instance, microbiome samples from an evidentiary item and a potential suspect would need to be processed on separate runs, regardless of unique dual-indexing mitigation strategies, to avoid any possibility of cross-contamination, which may arise during library preparation or sequencing [88]. Index-hopping could, therefore, be largely avoided between different forensic samples; however, batch effects may still persist for samples of the same evidentiary origin, limiting the potential forensic value of comparing reference (known origin) microbiome samples to questioned samples.

For forensic utility, multiple samples, potentially from the same item or several similar items, in addition to negative controls and unique dual-indexing strategies, would ideally be processed on a single run to reduce the associated time and costs involved with sequencing. However, this would still necessitate careful consideration and reliable computational approaches [102] to verify the origin of observed microbiota within a sequenced sample, to validate signals as ‘genuine microbiomes’ of interest and not artefacts of index-hopping or batch effects. Obtaining sufficient sample and processing replicates (i.e., of the same sample) may provide greater confidence in the identification of a microbial community of interest in comparison to a negative control; however this may be a challenging approach for forensic applications if the sample in question originates from trace evidence, in which starting quantities of extracted DNA will be low.

While there is no standard method for the analysis of 16S rRNA sequencing data, there is currently software frequently used that is freely available to users to process raw data, including parameters involving filtering, de-noising, taxonomic assignments and phylogenetic analyses; QIIME2 [55] is one such example. Bioinformatics analysis of microbiome data is unique due to the compositional nature of the data itself; microbiome data is highly sparse—typically containing many zero-values, indicating the absence of taxa [103], which makes it inappropriate for analysis using most established ecology-based statistical approaches. Apart from the obvious challenges of training forensic personnel to conduct bioinformatic analyses at an appropriate standard, there are also further unique challenges for forensic scientists concerning the bioinformatics component of microbial profiling. Given how rapidly the bioinformatic field is evolving, every implemented update within the software, or the introduction of a new, more appropriate, parameter, may expose the lack of validity for a previously processed and evaluated microbial profile. Take for instance the updated approach of taxonomic clustering; previously, sequences were clustered into Operational Taxonomic Units (OTUs) at a 97% similarity threshold for species identification. More recently, however, bioinformatic analysts have applied Amplicon Sequence Variants (ASVs), which effectively cluster sequences at 100% similarity, due to the argument that previous clustering thresholds were now considered ‘too low’ for accurate species identification [104,105]. For forensic purposes, if a previous analysis was demonstrated to be invalid due to an evolving bioinformatics approach, this could result in the identification of a miscarriage of justice or necessitate the review of previous cases using the outdated approach.

### 3.5. Training, Interpretation, Future Research and Recommendations

If microbial profiling were to be considered for forensic purposes, the methods and protocols utilised must first be validated and established as reliable; specific standards set by accreditation bodies must also be met for the validation of microbial profiling. It should also be noted that such accreditation bodies may actually develop new standards, specifically for microbial profiling. While microbial profiling issues have been highlighted [88], and recommendations have been made to elevate microbiome studies to a standardised level [54,94,98,106,107], no such specific standards exist for the forensic implementation of microbial profiling. However, it is worth noting that microbial forensics (traditionally involving bioterrorism), which received significant attention following the events of 9/11 in the US and the Bali bombings in 2002, was implemented as a program within some jurisdictions involving compliance testing by some national testing authorities [108]. Therefore, in a potential future involving the forensic application of microbial profiling, the involvement of agencies that set standards and accredit laboratories would be crucial to uphold high standards of admissibility of microbial profiles as forensic evidence.

Future research in this area must address factors such as extraction and sequencing sensitivities, specificity, microbial profile reproducibility, population frequencies of specific microbiota within human microbiomes, impacts of environmental factors, and incorporate mock casework relevant samples of known ground truth. Relevant variables and their level of impact on the outcome of an obtained microbial profile must also be comprehensively understood, as well as the limitations of applied methodologies. Following this, their application by forensic practitioners must be reliable, and may be assessed through proficiency testing. It is imperative that the provided training enables forensic practitioners to apply bioinformatic methods appropriately given set protocols to analyse data generated and interpret such data in the context of case-related questions. In addition to this, protocols would need to be established to record in detail the parameters applied, the software and/or plugin versions utilised and any manipulations to the data that may be considered subjective. For example, the selection of a sampling depth, also known as ‘rarefaction’, implemented for subsequent phylogenetic analysis, is a component that is considered data-dependent and is subjectively assessed [109]. The forensic utility of microbiome-associated informatics tools would need to be restrictive in terms of the types of analyses applied, and rigorously tested against mock samples of known microbial compositions and mock case work samples. These restrictions would safeguard against potential misidentifications, if the identification or association of individuals with criminal activities was the intended forensic application. The analysis of microbial data may also require established forensic databases of microbial taxonomy for the reliable identification of microbial species, since taxonomic identification differs greatly, depending on which database is applied to the data of interest [110,111].

In addition to these considerations, microbial forensics would require protocols for the reporting of analysis results and any resulting human identification or association of individuals using their microbiomes and associated error rates, to maintain consistency across national and international laboratories on reporting standards and ensure appropriate comprehension of the facts by laypersons. To this end, there would need to be further research to determine the probabilities for human-associated microbial transfer, and, thus, the associated probabilities for calculating the likelihood of the microbial DNA evidence, given activity level propositions, one of which may relate to innocent microbial transfer. Innocent transfer may be perceived as, for example, the deposition of millions of bacterial cells from individual’s microbial cloud into a built environment [112], which could later be established as a crime scene, or the indirect transfer of one individual’s microbiome to another individual through their presence within a shared space or handling of an item [34,69,76].

Further areas of research within microbial forensics, specifically involving the potential identification or association of individuals through the analysis of their own microbiomes and those of evidentiary items/crime scenes, should address the deconvolution of mixed microbial profiles. Given that a microbial profile does not include obvious indicators of mixed-source origins (i.e., microbiota originating from two or more individuals), profile deconvolution of potentially mixed-source signals would be an obvious, yet necessary, challenge to reliably lead forensic investigations. Some methodologies have been investigated, which attempt the deconvolution of mixed-source microbial profiles, including Random Forests classification models [113], in which the microbial similarity of samples is interrogated [34] and the microbial ‘owners’ of the profiles are predicted. One such study investigated the microbiomes of students sharing dormitory rooms using a Random Forests model, but demonstrated an increased error rate in profile deconvolution of mixed-source microbial samples when two or more students shared a room, even when the subjects were known and accounted for through personal sampling of their own microbiomes [34]. Understandably, and as the authors acknowledged [34], the lack of empirical support for this approach indicated a need to exercise caution for future studies involving mixed-source microbial profiling. Another approach, which appeared to have greater resolve, involved Bayesian prediction software, namely SourceTracker, which correctly attributed pubic mound taxonomic distributions of couples to their partners following close sexual contact, but only in instances where at least 10% of the individual’s pubic microbiome was derived from their partner; single transfer events were generally non-discoverable, limiting the potential forensic application of this approach [12]. Additionally, these studies involve microbiomes of known individuals, which further questions the applicability of microbial profiling for forensic utility for instances in which no suspect(s) have been identified, or when a sample is known to be of mixed-origin.

In a more recent study, prediction software was used to trace mock burglars’ interactions with surfaces within artificial crime scenes [114]; despite the success in associating the correct burglar with the microbiome of a sampled surface, the authors concede that the accuracy of detection was far weaker than would be required by accepted forensic standards. As such, microbial profiling via 16S rRNA amplicon sequencing, as it is currently performed, should not be considered reliable for forensic trace evidence [114]. In addition to the performance standards of this approach, future research should address how an individual’s microbial profile (in particular, the rare species, which may be used to differentiate individuals) is ‘unique’ or determined to be ‘unique’ to that individual. Furthermore, the authors of the mock burglary study state that absence of rare taxa in the microbiome of a sampled surface does not indicate a lack of interaction with that surface from an individual, i.e., an absence of evidence is not evidence of absence. However, we would argue that this be extended to consider the presence of rare, or distinguishing taxa within the microbiome of a sampled surface. The presence of specific taxa should not indicate a direct interaction with the surface from an individual who displays the same taxa within their microbial profile, given that indirect microbiome transfer between individuals has been previously assessed [69] and has been shown to occur in such settings, in addition to the indirect transfer of microbiomes from an individual’s microbial cloud into a built environment, which occurs passively [2]. Further research should not only address how individuals may be linked to rare, or distinguishing microbes within complex environments that may exhibit background microbiomes, but also whether the approach should at all be considered for forensic utility provided that an incorrect classification or interpretation of a microbial profile could seriously mislead forensic investigations. There is much information currently lacking in the research surrounding the utility of microbial profiling for forensic purposes. An important step forward would be to identify the real possibility of the challenges and limitations discussed in this review within an experimental setting, including the potential for microbial transfer and/or contamination to occur. Future studies involving these considerations may help to outline realistic instances where microbial profiling may add value to forensic investigation, given the capabilities of current highly discriminating and well validated forensic human DNA methodologies. Further research is required in multiple areas before human skin microbial profiling can be reliably applied to assist forensic investigation. This includes, but is not limited to:

(a) Determining the variability of profiles from deposits made by different areas of skin, within and between individuals, over time, and investigating factors impacting these differences;
(b) The impact of time and environmental conditions during the period between a deposit of the material of interest and the time of interest, and when the sample is collected;
(c) The efficiency of sampling and storage methods in terms of microbial DNA quantity and profile integrity;
(d) The assessment of contamination risks throughout the process from sampling through to profiling, and means of mitigation.

Therefore, the authors of such work should exercise restraint in overstating the potential advantages or forensic utility of microbial profiling until further studies have outlined potential limitations, and where possible, outline effective strategies to mitigate these limitations to the point that it is clear in which circumstances microbial profiles may be deemed reliable.

## 4. Conclusions

The forensic utility of microbial profiling for the identification and/or the association of individuals with criminal activities may be in its infancy regarding research and development, but the potential limitations noted above must be discussed and researched before contemplating its use in case work. An increased interest in microbiome research and genomic data analysis invites an increased interest toward the expansion of potential forensic applications [115,116,117,118]. If the challenges noted here are reflected upon more closely by the forensic community, as they have been examined across other fields concerned with microbiome research [9,54,88,94,119], then it may be argued that further research into the potential forensic applications of microbial profiling would see a shift in focus towards establishing the circumstances in which this approach could be used, rather than how it may be used.

## Figures and Tables

**Table 1:** Summary of the potential utility and advantages of microbial profiling, with associated limitations and challenges associated with using the human microbiome for forensic human identification and/or association via a 16S rRNA bacterial profiling approach.

Potential Utility/Advantages	Associated Limitations and Challenges
▪ Human microbiomes may be highly individualised. ▪ Human microbial profiling may assist investigation of criminal activities by providing information not available utilising current forensic methodologies.	▪ Human microbiomes can be transferred between people, both directly and indirectly. ▪ Unlike human DNA, human microbiomes differ among areas of a body, change over an individual’s lifetime, and are dependent on habits and environments occupied. ▪ Human DNA may degrade but its profile does not change when a forensically relevant sample is collected sometime after it has been deposited, while the human microbial profile may change over this period. ▪ Forensic human DNA profiles are made up of core sets of loci with alleles of known population frequency, while human microbial profile components are plentiful, variable, and their frequency and proportions within sample types are relatively unknown.
▪ Human-associated microbiota are in high abundance compared to human somatic cells, and may be more readily available from crime scenes than human DNA. ▪ Microbial profiles can be produced from low-biomass samples, which may be useful for trace evidence.	▪ A sample of almost anything (e.g., surfaces, items, equipment, reagents) can produce a microbial profile, potentially compromising the interpretability of the microbial profile of the target sample. ▪ As human microbiomes can be altered or change over time, the lapse in time between microbial sample collection from a crime scene and microbial sample collection from potential suspect(s) may impact comparisons. ▪ It must be demonstrated that microbial profiles of low-biomass samples are different from profiles of control samples. Negative extraction controls often return non-negative results and low-biomass samples are often impacted by exogenous microbial contamination.
▪ Human-associated microbial communities are driven by body-site differences.	▪ Identifying body sites associated with a sample may only be useful for investigations in which potential suspect(s) have been identified. ▪ If no suspect(s) have been identified, it may be challenging to determine if a microbial sample, and the associated profile, is of mixed-origin.
	▪ Microbial contamination can occur throughout sample collection to sequencing and may originate from multiple technical and environmental sources.
	▪ Microbial forensics may require new standard operating procedures to collect, store and extract microbiome samples. These new procedures may not be compatible with existing procedures designed and optimised for human nDNA recovery.
	▪ Microbial profiles may be difficult to reproduce, especially if a different collection protocol, DNA extraction kit, technician or environment (i.e., forensic laboratory setting) is applied.
	▪ Current recommendations for microbiome studies to reduce variation, including sequencing batch effects, may not be suitable for forensic protocol, and for microbial profiling of evidentiary and questioned samples.
	▪ Bioinformatics is constantly evolving, which may influence the outcome of microbial profiles and thus hinder valid comparisons of profiles determined by different tools.
	▪ Forensic practitioners are by and large unfamiliar with the methodologies and procedures of generating, interpreting and reporting microbial profiles, thus a significant investment in training and authorisation would be required.
